# A Virus-Free Poly-Promoter Vector Induces Pluripotency in Quiescent Bovine Cells under Chemically Defined Conditions of Dual Kinase Inhibition

**DOI:** 10.1371/journal.pone.0024501

**Published:** 2011-09-02

**Authors:** Ben Huang, Tong Li, Lucia Alonso-Gonzalez, Ruben Gorre, Sarah Keatley, Andria Green, Pavla Turner, Prasanna Kumar Kallingappa, Vinod Verma, Björn Oback

**Affiliations:** 1 AgResearch, Ruakura Research Centre, Hamilton, New Zealand; 2 Animal Reproduction Institute, Guangxi University, Nanning, China; 3 Children's Cancer Research Group, Department of Paediatrics, University of Otago, Christchurch, New Zealand; 4 Trenzyme GmbH, Konstanz, Germany; 5 Department of Biochemistry, National University of Singapore, Singapore, Singapore; University of Sao Paulo–USP, Brazil

## Abstract

Authentic induced pluripotent stem cells (iPSCs), capable of giving rise to all cell types of an adult animal, are currently only available in mouse. Here, we report the first generation of bovine iPSC-like cells following transfection with a novel virus-free poly-promoter vector. This vector contains the bovine cDNAs for *OCT4, SOX2, KLF4* and *c-MYC,* each controlled by its own independent promoter. Bovine fibroblasts were cultured without feeders in a chemically defined medium containing leukaemia inhibitory factor (LIF) and inhibitors of MEK1/2 and glycogen synthase kinase-3 signaling (‘2i’). Non-invasive real-time kinetic profiling revealed a different response of bovine vs human and mouse cells to culture in 2i/LIF. In bovine, 2i was necessary and sufficient to induce the appearance of tightly packed alkaline phosphatase-positive iPSC-like colonies. These colonies formed in the absence of DNA synthesis and did not expand after passaging. Following transfection, non-proliferative primary colonies expressed discriminatory markers of pluripotency, including endogenous iPSC factors, *CDH1*, *DPPA3*, *NANOG, SOCS3, ZFP42,* telomerase activity, Tra-1-60/81 and SSEA-3/4, but not SSEA-1. This indicates that they had initiated a self-sustaining pluripotency programme. Bovine iPSC-like cells maintained a normal karyotype and differentiated into derivatives of all three germ layers in vitro and in teratomas. Our study demonstrates that conversion into induced pluripotency can occur in quiescent cells, following a previously undescribed route of direct cell reprogramming. This identifies a major species-specific barrier for generating iPSCs and provides a chemically defined screening platform for factors that induce proliferation and maintain pluripotency of embryo-derived pluripotent stem cells in livestock.

## Introduction

Pluripotent stem cells (PSCs) are capable of unlimited proliferation in vitro and generation of all adult cell types, including functional gametes. They are either derived from a transient cell population in the inner cell mass of pre-implantation blastocysts (embryonic stem cells or ESCs) [1], [2] or from post-natal testis [3]. More recently, PSC-like cells were also derived from post-implantation egg cylinder embryos [4], [5]. These epiblast stem cells (EpiSCs) express the core transcriptional pluripotency network [6], [7], [8] and are capable of multi-lineage differentiation [4], [5]. However, ESCs and EpiSCs represent discrete pluripotent states, termed ‘naïve’ and ‘primed’, respectively [9]. In contrast to naïve ESCs, primed EpiSCs show flattened rather than packed dome morphology. They have predominantly undergone X-chromosome inactivation in female cells, up-regulated certain specification markers (e.g. *Fgf5, Lefty2, T* or *T-brachyury*) and down-regulated others that distinguish them from ESCs (e.g. *Dppa3* or *Stella, Zfp42* or *Rex1, Nr0b1*, *Klf2, Stat3, Lifr, Stat3 and Socs3*) [10], [11], [12].

Functionally, EpiSCs are poised to differentiate into primordial germ cells in vitro and neither contribute to all somatic cell lineages nor the germline in chimeras [13]. Under chemically defined conditions, murine ESCs will self-renew or differentiate, respectively, in response to leukemia inhibitory factor (LIF) and fibroblast growth factor (FGF)/extracellular signal-regulated kinase (ERK)-signaling [14]. In contrast, EpiSCs do not respond productively to LIF and can be stably propagated in the presence of FGF and activin [4]. Upon FGF/activin withdrawal and ectopic expression of either *Klf2*, *Klf4* [10], *Nanog* [15] or *Nr5a* [16], EpiSCs can robustly revert to naïve ‘ground state’ pluripotency.

Delivering ectopic pluripotency-inducing genes into somatic cells has become an established route of generating fully pluripotent cells (induced pluripotent stem cells or iPSCs) by genetic manipulation [17]. iPSCs can be identified by morphological, molecular and functional criteria [18], [19], [20], [21], [22]. Completely reprogrammed iPSCs show mRNA and microRNA expression patterns that are highly similar to isogenic ESCs [23]. DNA demethylation of the *Oct4* (or *Pou5f1*) and *Nanog* promoter regions and global patterns of histone methylation are virtually indistinguishable from ESCs [19], [21], [22], [24], [25]. Functionally, genuine iPSCs contribute to the germline in chimeric mice [22], [26], [27] and support the development of embryos entirely derived from iPSCs [28], [29], [30]. These functional assays have become the most stringent criteria to define naïve pluripotency [31].

Somatic cells from mouse [17], rat [32], [33], pig [34], [35], sheep [36], horse [37], rabbit [38], monkey [39], [40], and human [41] have been reprogrammed using different factor combinations, including *OCT4 (O)*, *SOX2 (S)*, *KLF4 (K)*, *c-MYC (M)*, *LIN28*, *NANOG* [41] or *Nr5a2* replacing *OCT4* [42]. Factors are usually delivered into cells by integrating viral vectors [18], [41], [43], but integration-free reprogramming has also been achieved [44], [45], [46], [47], [48], [49]. To stabilize signaling pathways that maintain pluripotency, culture media can be supplemented with small molecules that alleviate differentiation cues. Ying et al. cultured murine ESCs in medium that relies on the double inhibition (‘2i’) of mitogen-activated protein kinase kinase (MAP2K1/2 or MEK1/2) by PD0325901 and glycogen synthase kinase 3 beta (GSK3B) by CHIR9902, respectively, to effectively promote pluripotency [14]. Together with the self-renewal cytokine LIF, which directly improves reprogramming efficiency [12], 2i/LIF medium enabled the conversion of mouse EpiSCs into ESCs [10] and from partially into fully reprogrammed iPSCs [50]. It also led to the first derivation of bona fide ESCs from rat embryos [51], [52]. Notably, application of 2i/LIF helps establishing a naïve or ‘mouse ESC-like’ pluripotent state in human embryonic stem cells and human iPSCs [11].

Over the past three decades, it has been difficult to derive PSCs from other mammalian species and all attempts to derive chimera-competent bovine PSCs have failed [53], [54]. Here we explore the possibility that 2i/LIF promotes pluripotency in cattle, a species previously considered non-permissive for PSC-derivation [50].

## Results

### Bovine iPSC-like colonies form in 2i/LIF medium

**Figure 1:**
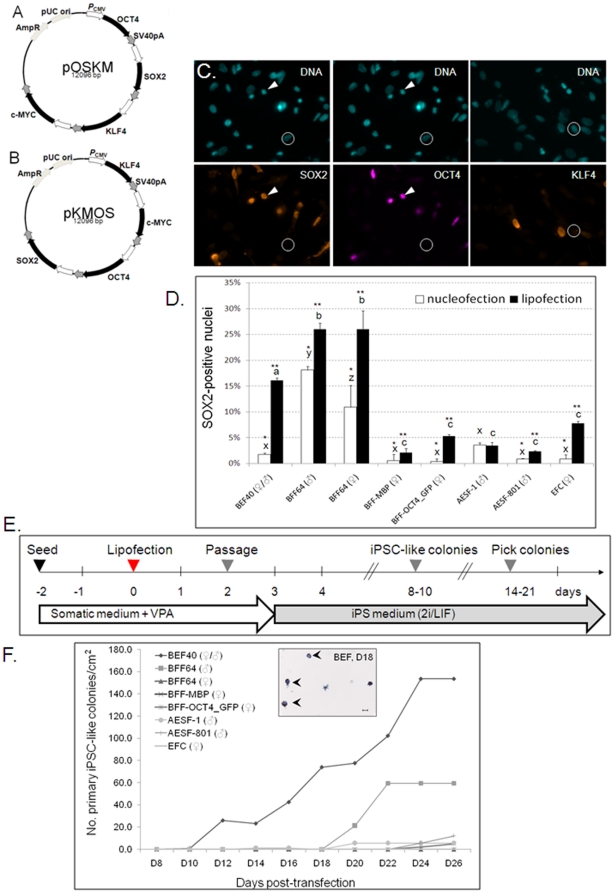
Generating bovine iPSC-like cells. (A, B) Maps of poly-promoter constructs used in this study; (C) Immunofluorescence 24 h post-transfection with pOSKM. DNA was counterstained with Hoechst 33342. OCT4 and SOX2 co-localized in nuclei (arrowheads), circled cells stained negative; (D) Transfection efficiency; bars with different superscripts differ *P*<0.05 for lipofection (^abc^), nucleofection (^xyz^) or across groups (*, **); (E) Timeline of iPSC generation using lipofection. (F) iPSC-like colonies appear after pOSKM lipofection. Insert: AP+ BEF40-derived colonies (arrowheads), 18 days post-transfection. Scale bar  = 100 µm.

In order to deliver all four factors into the same cell, we constructed poly-promoter plasmids containing the complete bovine cDNAs for *OCT4, SOX2, KLF4,* and *c-MYC* (Fig. 1A, B). We first optimized several parameters for inducing pluripotency. Eight different cell lines (embryonic [1 line] vs fetal [4 lines] vs adult [2 lines] fibroblasts vs follicular cells [1 line]) were transfected (lipofection vs nucleofection) with two different plasmids (pOSKM vs pKMOS). SOX2, OCT4, and KLF4 showed appropriate nuclear localization after transient transfection (Fig. 1C). Following passaging and re-plating after 48 h, SOX2 immunostaining showed that embryonic and fetal fibroblasts transfected most efficiently (16% and 26%, respectively, Fig. 1D). Lipofection was generally superior with no significant differences between the two plasmids (data not shown). We then passaged lipofected cells onto laminin-coated plates and replaced the somatic with 2i/LIF medium (Fig. 1E). Dome-shaped, tightly packed colonies with clear borders started to appear around 10 days post-transfection and continued to increase in size and number thereafter (Fig. 1F). With the exception of BFF-MBP, all cell lines gave rise to colonies (Fig. 1F). Transfection efficiency correlated well with colony formation (r^2^ = 0.9591). We observed no significant differences between the two plasmids and only a small number of BEF40-derived colonies after nucleofection (data not shown). Since BEF40 resulted in the highest yield of colonies, we concentrated on this line for subsequent experiments.

**Figure 2:**
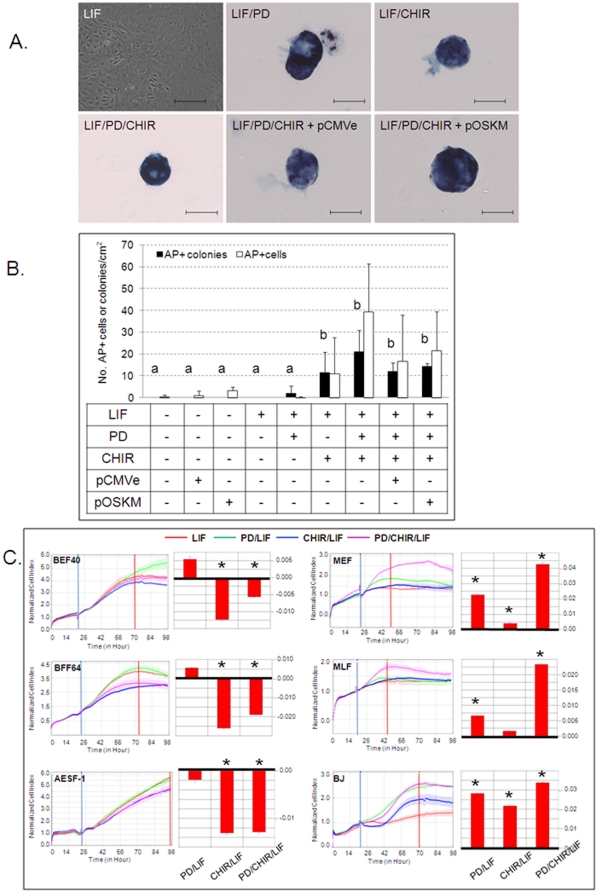
2i/LIF affects colony formation and AP induction. (A) AP+ colonies after no, empty vector (pCMVe) or pOSKM-transfection. Scale bar = 100 µm; (B) Quantification of AP+ iPSC-like colonies, 15 days after passaging BEF40; ^ab^ treatments differ P<0.005 for colonies. (C) xCELLigence™ real-time kinetic profiling. Cell indices were determined for bovine and murine embryonic (BEF40, MEF, respectively), bovine fetal (BFF) and bovine, murine and human adult fibroblasts (AESF, MLF, and BJ, respectively). Curves were normalized after compound addition (blue vertical line) and slopes (red bars) determined during log phase (between blue and red vertical lines); * =  slopes differ P<0.05 from LIF-treated control.

Following transduction, 2i/LIF medium has been shown to promote reprogramming into naïve pluripotency in both mouse and human iPSCs [11], [50]. We therefore cultured mock- or pOSKM-transfected cells in N2B27 supplemented with or without LIF, PD or CHIR. PD applied alone or in combination with CHIR and LIF greatly decreased phospho-MEK1/2 levels, while total MEK1/2 protein was not affected (Fig. S1). CHIR alone did not modulate phospho-MEK1/2. BEF40 cultured in either N2B27 or N2B27/LIF retained the typical morphology of bovine fibroblasts serum-starved into quiescence [55] (Fig. 2A). Addition of PD or CHIR to non-transfected cells, either with or without LIF, was sufficient to induce formation of colonies, of which 77±4% were AP+ (Fig. 2A). These colonies were in size, morphology and AP staining intensity indistinguishable from transfected colonies cultured under the same conditions (Fig. 2A). The average area and number of nuclei per AP+ colony on D16, determined from confocal sections of Hoechst-stained samples (n = 14), was 47429±2839 µm^2^ and 156±21, respectively. iPSC-like cell nuclei were significantly smaller than their ancestral BEF40 nuclei (23±4 µm^2^ vs 131±15 µm^2^, P<0.001). The number of CHIR-induced AP+ single cells and colonies was higher than for non-supplemented N2B27 or PD alone but not significantly different from cells treated with PD/CHIR or PD/CHIR + pOSKM (Fig. 2B). PD also induced AP+ colonies but this was not significant (P = 0.20) compared to non-supplemented medium. Culture in 2i/LIF was necessary to induce AP activity, as pOSKM-transfected BEF40 in N2B27 did not give rise to AP+ colonies. When we cultured non-transfected murine embryonic or human skin fibroblasts in LIF, PD, and CHIR, alone or in combination, we saw no morphological transformation into colonies or AP induction (n = 4 independent experiments). Overall, we transfected 2.35×10^4^ cells/cm^2^ with a transfection efficiency of approximately 16% ( = 3.76×10^3^ transfectants/cm^2^) and obtained about 15 AP+ colonies/cm^2^, resulting in a reprogramming efficiency of 0.4%.

To better characterize the response of bovine vs comparable murine and human cell types to 2i/LIF, we conducted non-invasive kinetic cell profiling using the xCELLigence™. This integrated real-time system displays changes in cell proliferation, viability, morphology and adhesion as CI values. In bovine, addition of LIF, PD, or PD/LIF had no significant effect compared to non-treated controls. After normalization on LIF-treated controls, both CHIR and PD/CHIR significantly reduced the CI in different bovine cell types (Fig. 2C). In contrast, PD, CHIR and PD/CHIR all significantly increased the CI in both murine and human fibroblasts (Fig. 2C).

### Bovine iPSC-like colonies do not expand in 2i/LIF

**Figure 3:**
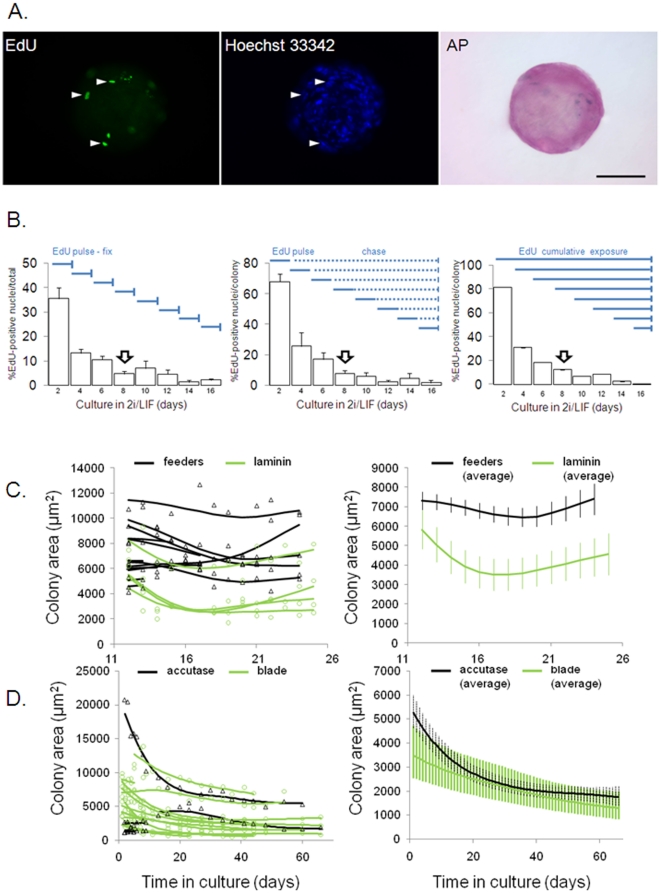
Bovine iPSC-like colonies do not expand in 2i/LIF. (A) Primary AP+ colonies with EdU+ nuclei (arrowheads) after 24 h label. Scale bar  = 50 µm. (B) DNA-synthesis after EdU addition (solid blue horizontal lines). EdU+ nuclei were counted after fixation (solid blue vertical lines). Open arrow indicates first emerging colonies. (C) Prior to passaging, colonies from feeders (black triangles) or laminin (green circles) were tracked. Regression splines were plotted for each colony (left) and averages (right graph). (D) After accutase (black triangles) or blade (green circles) passaging, colonies on laminin were tracked. Regression splines were plotted for each colony (left) and averages (right graph).

Next we investigated the mechanism of bovine iPSC-like colony formation. As a proxy for cell proliferation, we quantified DNA-synthesis following different EdU-incorporation protocols. Only AP+ colonies were quantified (Fig. 3A). EdU was added to each transfected culture on D0, 2, 4, 6, 8, 10, 12 or 14 following the addition of 2i/LIF and cells fixed 48 h after labelling (‘pulse-fix’, Fig. 3B). When the first colonies become visible, only 5±1% of cells still synthesized DNA and this further declined to 2±0.6% after two weeks in culture. To determine what proportion of EdU-incorporating nuclei became part of iPSC-like colonies, cells were labelled every two days (‘pulse’), washed out of EdU (‘chase’) and further cultured until fixation on D16 (‘pulse-chase’, Fig. 3B). After a pulse during the first two days in 2i/LIF, 68±12% of nuclei within each colony ended up being labelled with EdU on D16. When pulsed around the time of colony formation (D8), only 8±2% of cells within each colony still synthesized DNA. This proportion further declined to 2±2% after two weeks in culture, closely matching results from pulse-fix experiments. In order to detect slowly cycling cells, cultures were continuously kept in EdU until fixation at D16 (‘cumulative label’, Fig. 3B). This demonstrated that most cells in colonies (81±0.02%) were cycling at least once during the culture period. However, during the time of primary colony formation only 10% of cells within each colony still synthesized DNA and, in agreement with the pulse-label experiments, colonies labelled towards the end of the culture period (>D12) contained less than 5% proliferating cells. Cumulatively labelled non-transfected control cells, cultured in either 2i/LIF or N2B27, also became non-proliferative over time (Fig. S2A). This indicates that culture conditions, not plasmid-induced reprogramming, induced quiescence. Using immunofluorescence, we further quantified the proportion of cells expressing cell proliferation markers Ki-67 and proliferating cell nuclear antigen (PCNA) in pKMOS-transfected BEF40 cells (Fig. S2B) and colonies (Fig. S2C). For both antigens, the proportion of positive cells progressively declined over time (Fig. S2D). Using the Click-iT EdU assay, the proportion of apoptotic cells in primary colonies was small (<5% of total nuclei), indicating that most bovine iPSC-like cells in 2i/LIF were neither proliferating nor apoptotic but quiescent (Fig. S2E). Taken together, these results suggest that colonies primarily formed from non-proliferating cells and did not expand further through cell division.

Once colonies had formed, we determined whether their growth was influenced by the culture substrate. Colonies were manually picked and plated on feeder cells or laminin. Individual colonies were tracked for two weeks and their area was determined at regular intervals (Fig. 3C). All tracked colonies stained AP+ at the end of the tracking period. On both substrates there was no significant increase in colony area (n = 13 and n = 4 for feeders vs laminin, respectively). Regression splines plotted for each colony and colony averages showed no significant differences in shape (P = 0.2), indicating that colony growth was not differentially affected by substrate composition.

In order to establish bovine iPSC lines, we passaged colonies using either accutase or microblade dissociation. Initial plating efficiency was high (20/22 = 91% vs 22/22 = 100%, respectively). After first passaging, colonies growing on laminin were tracked for 65 days and their area determined (Fig. 3D). Tracked colonies stained AP+ at the end of the tracking period. In all cases (n = 5 and n = 12 for accutase vs blade, respectively), colony area significantly declined over time (P<0.001). Regression splines plotted for each colony and colony averages showed no significant differences in shape (P = 0.13), indicating that colony growth was not differentially affected by the passaging regime. In summary, colonies that formed from quiescent cells in 2i/LIF showed no signs of expansion for extended periods of time after passaging.

### Bovine iPSC-like colonies express discriminatory markers of pluripotency

**Figure 4:**
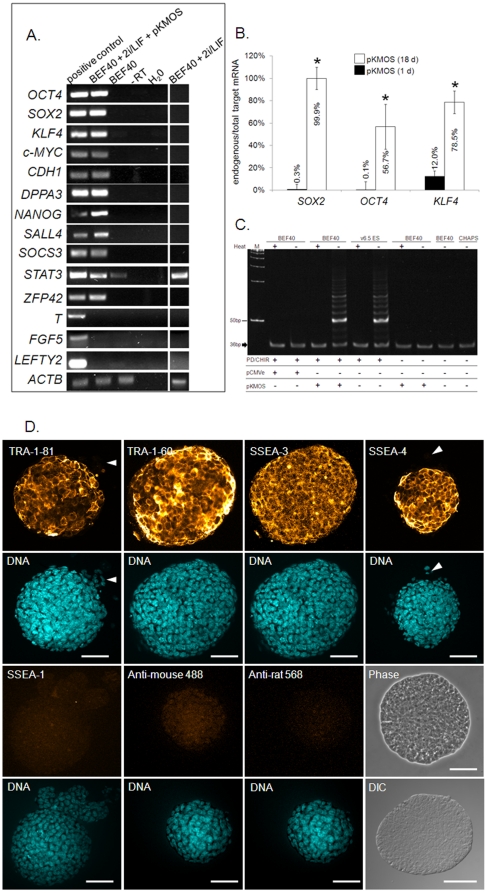
Molecular characterization of bovine iPSC-like colonies. (A) RT-PCR analysis. cDNA was extracted from 10 colonies/pool, cDNA from 10 bovine blastocysts/pool (bovine D16 epiblasts and MII oocytes for *T-BRACHYURY* and *FGF5*, respectively) provides a positive control. (B) qRT-PCR of endogenous vs total target gene expression in cells vs colonies (1 d vs 18 d post-transfection, respectively); target genes values were normalized on *18S* expression. * =  values differ P<0.05 between time points; (C) Telomerase activity using the TRAP assay. Heat-inactivated (+) samples and CHAPS buffer serve as negative, mouse ESCs (v6.5) and 36 bp internal control band (arrow) as positive controls. (D) Confocal immunofluorescence. DNA was counterstained with Hoechst 33342. Arrowheads indicate negative cells. Stainings without primary antibodies (anti-mouse 488 and anti-rat 568) provide negative controls. Phase  =  phase contrast, DIC  =  Differential interference contrast. Scale bar  = 50 µm.

We then evaluated molecular markers of pluripotency. Culturing BEF40 in somatic medium or 2i/LIF did not induce detectable expression of pluripotency-associated transcription factors (Fig. 4A). In contrast, pKMOS-transfected BEF40 in 2i/LIF induced *NANOG*, *SALL4* and *DPPA3*, while markers of primed EpiSCs (*FGF5, LEFTY2, T-BRACHYURY*) were not detectable (Fig. 4A). Using primers that distinguished between endogenous (Fig. S3A) and ectopic iPSC factors (Fig. S3B), we determined that one day post-transfection most *SOX2*, *OCT4* and *KLF4* transcripts were of ectopic origin (99.7%, 99.9% and 88%, respectively), whilst in primary colonies (18 days post-transfection), most transcripts were transcribed from endogenous loci (99.9%, 56.7% and 78.5%, respectively, Fig. 4B). Using plasmid-specific primers, we detected presence of the transgene in genomic DNA of D38 colonies, indicating stable integration of the vector (Fig. S3C). Telomerase activity, evident by a ladder of products with 6 base increments, was high in bovine iPSC-like cells and mouse ESCs used as a positive control (Fig. 4C). Neither non- or mock-transfected BEF40 in 2i/LIF, nor pKMOS-transfected BEF40 without 2i/LIF showed detectable telomerase activity. Confocal immunofluorescence analysis showed that iPSC-like cells expressed the pluripotency surface markers SSEA-3/4 and TRA-1-60/81, but not SSEA-1 (Fig. 4D). They also expressed OCT4 and SOX2 (Fig. S4). None of these markers were detected in non-transfected BEF40 in 2i/LIF (data not shown). Taken together, these data show that bovine iPSC-like colonies displayed several discriminatory markers of pluripotency.

### Bovine iPSC-like colonies differentiate in vitro and in teratomas

**Figure 5:**
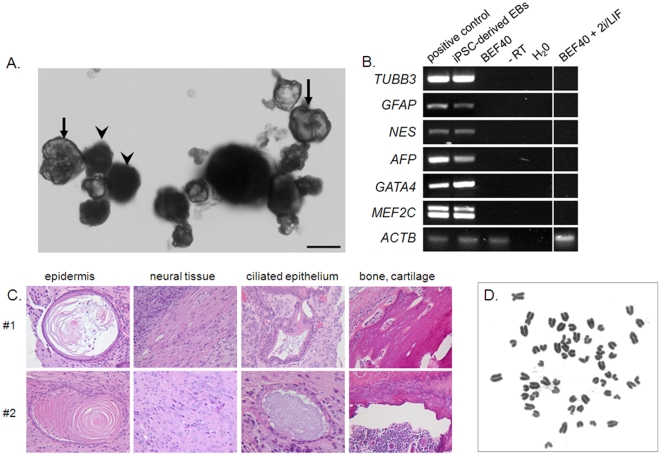
Differentiation of bovine iPSC-like colonies in vitro and in teratomas. (A) Simple (arrowheads) and cystic (arrows) EBs. Scale bar  = 50 µm. (B) RT-PCR analysis of differentiation marker expression in cystic EBs. cDNA from bovine fetal brain (ectoderm), gut (endoderm) and heart (mesoderm) provides a positive control. (C) Histological sections of intramuscular teratomas from different pools of colonies (#1, #2). (D) Karyotype of bovine iPSC-like cells after 24 days in culture.

Bovine iPSC-like colonies formed solid and cystic EBs after 5 days to 3 weeks, respectively, in N2B27 suspension culture (Fig. 5A). These expressed ectoderm- (*TUBB3, GFAP, NES*), endoderm- (*AFP*) and mesoderm- (*GATA4, MEF2C*) markers (Fig. 5B). Following injection of iPSC-like cells from two independent tranfections into SCID mice, large tissue masses, ranging from 8–20 mm in diameter and 1–5 g in weight, were harvested after seven weeks from two out of four hind legs. Histological examination of the two specimens showed differentiation into ectoderm (epidermis, neural tissue), endoderm (ciliated epithelium) and mesoderm (bone, cartilage) (Fig. 5C). These features were consistent with intramuscular grade 3 teratomas. Bovine-specific primers detected *ACTB* in both genomic DNA (Fig. S5A) and reverse transcribed cDNA (Fig. S5B) from bovine iPSC-, but not murine ESC-derived, teratomas. This confirms that the tumor originated from bovine cells. We conclude that iPSC-like cells are capable of multi-lineage differentiation and production of complex teratomas. Lastly, we karyotyped bovine iPSC-like cells after 24 days in culture and all cells (n = 12/12) showed a normal number of 60 chromosomes (Fig. 5D).

## Discussion

Here we report the first reprogramming of non-proliferating bovine cells into pluripotency under conditions of chemically defined signal inhibition. We refer to these cells as “iPSC-like” because their capacity for germline chimerism remains to be demonstrated. The first iPSC reports used integrating viruses to carry the pluripotency genes into cells, potentially disrupting endogenous genomic information and causing tumors [56]. Episomal viruses reduce the risk of insertional mutagenesis during iPSC generation [45], [57], [58]. However, viral DNA may still trigger the immune system [59]. This raises biosafety concerns for agricultural applications where the products (e.g. meat, milk) of iPSC-derived farm animals or their offspring ultimately enter the human food chain. Our constructs avoid lentiviral backbones or foot-and-mouth disease virus 2A oligopeptides [46], [60], [61] that in many countries would not be permissive for commercial livestock applications. Instead, we used single transfection of a novel expression vector where each bovine iPS factor was flanked by its own independent CMV promoter. Since our work in bovine started, iPSCs in other species have been produced using plasmid-mediated approaches. This either involved serial co-transfection of two plasmids containing different sets of factors [44], [46], [49] or transfection of a polycistronic vector transcribing several factors from a single promoter [44], [49], [61]. In some cases, transfection approaches have resulted in iPSCs with no evidence of transgene integration [46], [61]. As primary bovine iPSC-like colonies were likely of mixed origin, i.e. derived from more than a single reprogrammed fibroblast, and generation of clonal cell lines was unsuccessful, we did not attempt to identify non-integrative iPSC clones. Presence of the transgene in genomic DNA of D38 colonies indicated that at least some cells had stably integrated the vector. Compared to virus-mediated delivery methods, plasmids are technically simple and relatively cheap, eliminating the need for specialized biohazard containment facilities to produce viral stocks [21]. Overall, bovine reprogramming efficiency, corrected for transfection efficiency, was 0.4%. This was consistent with virus- and plasmid-mediated approaches (ranging from 0–8% and averaging 1% [44], [46], [48], [62]). It remains to be determined if the new poly-promoter design and use of isogenic bovine sequences results in higher reprogramming efficiency of bovine cells than using alternative vector designs carrying homologous mouse or human iPS genes.

Following transduction, 2i medium promotes reprogramming into naïve pluripotency by neutralizing inductive differentiation stimuli in both mouse and human iPSCs [11], [50]. This effect appeared even more pronounced in bovine cells. CHIR and, to a lesser extent, PD induced colony formation and AP activity in bovine, but not murine or human, fibroblasts. How these two compounds elicit the transformation from single cells to compacted colonies, including changes in cell shape and size, migration and intercellular clustering, is not clear. It would be important to better characterize the dependency of bovine cells on MEK, GSK3B and components of their respective signaling pathways. Likewise, the signaling cascade leading to AP induction in bovine cells is not known. AP induction was unrelated to the morphological and molecular changes that caused colony formation, as it also occurred in single cells. High level of AP expression is a fairly non-specific marker for PSCs [63] that appears very early during the iPSC reprogramming [43]. More stringent molecular markers of pluripotency, such as telomerase activity, *NANOG*, and *SALL4,* were only induced when the cells were transfected with iPS vectors in the presence of 2i/LIF. This also applied to *DPPA3, SOCS3 and ZFP42,* all discriminatory markers of naïve pluripotency [10]. Silencing ectopic gene expression and activating endogenous iPS factors is considered another hallmark of full reprogramming [64]. We observed that *SOX2*, *OCT4* and *KLF4* transcripts predominantly originated from the endogenous loci. This indicates progressive epigenetic silencing of the CMV promoter, as previously described after transient transfection of ESCs [65]. Cell surface antigens SSEA-3/4 and TRA-1-60/81 also appear late during reprogramming and are considered among the most definitive markers of fully reprogrammed iPSCs [64]. Bovine iPSC-like cells strongly expressed both marker sets, similar to undifferentiated iPSCs in pig [35], monkey [39], [40], and human [41] but different from mouse [17] and rat [32], [33] which express SSEA-1 instead. Lastly, diagnostic markers of human embryo-derived stem cells and rodent EpiSCs (*FGF5, T-BRACHYURY,* and *LEFTY2*) were not detectable in 2i/LIF-cultured bovine iPSC-like cells, providing additional molecular evidence of reprogramming into pluripotency.

A molecular link between pluripotency and the capacity for unlimited self-renewal is the presence of telomerase. This ribonucleoprotein is specifically active in immortal cells, such as cancer, germ cells and PSCs [66], [67], stabilizing telomere length and extending cellular life span [68]. Bovine iPSC-like cells exhibited telomerase activity similar to mouse ESCs, suggesting that they were poised for long-term proliferation. The formation of large solid teratomas from a few thousand injected colonies indicates that bovine iPSC-like colonies did not irreversibly lose their proliferation potential in 2i/LIF and can resume cell division in the right environment.

Under serum-free 2i/LIF conditions, ERK-reliant cell types (e.g. MEFs) either die or become quiescent [50]. Murine and human iPSCs, on the other hand, arise from rapid proliferation [62], [69], [70]. In fact, it has been suggested that proliferation promotes pluripotency induction, whereas cell cycle arrest inhibits reprogramming and induces irreversible differentiation [70]. In mouse and human, individual reprogrammed cells first start to divide faster, getting smaller in the process and then giving rise to primary colonies through symmetric cell divisions [62], [69], [70]. As primary colonies expand, cells detach and form secondary colonies elsewhere [62]. Consequently, the number of primary colonies remains constant after some time, while the number of secondary colonies continues to increase [62]. We also observed a reduction in nucleus size as fibroblasts converted into iPSC-like cells, most likely during the first few days in 2i/LIF when most cells were still cycling. However, several lines of evidence suggest that proliferation was unlikely to play a major role during colony biogenesis: i) 95% of cells did not synthesize DNA when colonies formed (EdU pulse-fix); ii) >90% of cells present in colonies on D16 were outside S-phase one week earlier, around the time of colony formation (EdU pulse-chase); iii) >90% of cells within the population did not synthesize DNA during the week following colony formation (EdU cumulative labelling); iv) the proportion of Ki-67 and PCNA expressing cells decreased over time, correlating well with the decline in EdU-incorporation; v) primary colonies did not expand for several weeks before or after passaging; vi) the number of primary colonies stabilized after some time with no subsequent multiplication into secondary colonies. Irrespective of the EdU labelling protocol, <2% of cells still synthesized DNA in D16 colonies. At that time, almost all cells within each colony were non-apoptotic and positive for SSEA-3/4 and TRA-1-60/81, providing direct evidence for the quiescence of molecularly reprogrammed iPSC cells. The kinetic response of bovine fibroblasts vs their murine and human counterparts was also characterized using an xCELLigence™ electronic cell sensor array. Based on their reduced CI values, bovine cells significantly diverged from mouse/human in their overall cell proliferation, morphology, and/or adhesion response to PD and CHIR, but not LIF, addition. This provides further evidence for previously unidentified differences in signaling pathways between these species. Varying PD and CHIR concentration may alleviate their anti-proliferative effect, perhaps through minimizing side effects on other kinases. Excluding cell proliferation as the main mechanism of colony formation, bovine iPSC colonies in 2i/LIF likely formed through migration and aggregation of individual cells that had become quiescent before or during re-acquisition of pluripotency. It is conceivable that quiescence may have even enhanced cell reprogrammability, similar to its beneficial effect in nuclear transfer-induced epigenetic reprogramming [71], [72].

Several applications await bovine iPSC-like cells. First, they provide a chemically defined screening platform for candidate factors that maintain both proliferation and pluripotency of pluripotent stem cells in livestock. This is particularly relevant for establishing embryo-derived stem cells. Second, they may be converted into animals by using them as donors for somatic cloning with the prospect of significantly increasing cloning efficiency compared to conventional donors [73], [74]. Bovine iPSC-like cells will also be tested for their ability to generate germline chimeras. Since germline transmission of superior genetics is the most important criterion for breeding, such animals would serve the same purpose as clones. Third, the pluripotent cell state facilitates transgenesis and homologous recombination [75], [76], [77]. Provided their proliferation block can be overcome, bovine iPSC-like cells would facilitate the precise genetic engineering of farm animals for improved production traits and biopharming. Beyond these agricultural applications, bovine iPSC-like cells would help to provide large animal models for human diseases [78], [79], [80], [81], [82], [83], [84], complementing research currently carried out with laboratory animals.

## Materials and Methods

### Plasmid construction

Complete protein coding cDNA sequences of bovine *OCT4*, *SOX2*, *KLF4* and *c-MYC* were obtained from GeneBank (accession numbers NM_174580, NM_001105463.1, BC134523, and NM_001046074, respectively). After introducing silent mutations in *KLF4* (TCG to TCC at position 1398) and *OCT4* (GGT to GGA at position 967) and adding a 5′- *Xho*I and 3′-*Sal*I site, respectively, the altered sequences were synthesized (GENEART, Germany). The CDS encoding a zinc finger nuclease was removed from pRK5.GZF1-N and the plasmid ligated using a linker (Fw 5′-GGCTAGCTCGAGACGTG-3′, Rv 5′- GTCGACACGTCTCGAGCTAGCCGC-3′) that added an *Xho*I site downstream of the CMV promoter. The resulting plasmid was linearized with *Sac*I, and ligated using a linker (Fw 5′- CCTAGGGTACCACGTGAGCT -3′, Rv 5′- CACGTGGTACCCTAGGAGCT-3′) that added a *Kpn*I site upstream of CMV promoter (‘pCMVe-K). Following *Xho*I and *Sal*I digest, the four factors were each inserted into pCMVe-K, resulting in single-factor plasmids (pO, pS, pK, pM). pO and pK were digested with *Kpn*I, and the fragment containing both CMV promoter and vector sequence cloned into *Kpn*I-linearized pS and pM, respectively, resulting in double-factor vectors (pKM, pSO). Both were partially digested with *Kpn*I to isolate one fragment containing the vector, and one containing the two transcription factors with their CMV promoter. The fragments containing two factors were ligated into the linearized plasmids containing the other two, resulting in four-factor vectors (pOSKM, pKMOS). Plasmids were isolated using a PureLink™ HiPure Plasmid Filter Kit (Invitrogen).

### Cell culture and plasmid transfection

Primary bovine cell lines were isolated as described [85], [86]: hypodermal fibroblasts from pooled day (D) 40 male and female IVF embryos (‘BEF40’); fetal lung fibroblasts from a D64 female fetus and its male sibling (‘BFF64’); clonal cell strains of transgenic BFFs (‘BFF-MBP [87]’, ‘BFF-OCT4_GFP’ [88]); adult ear skin fibroblasts from two different bulls (‘AESF-1’, ‘-801’); and adult follicular cells (‘EFC’). Mouse lines were either derived from D13.5 embryos (‘MEF’) or adult lung tissue (‘MLF’). Human skin fibroblasts (‘BJ’) were obtained from ATCC® (CRL-2522™). In pilot experiments, mitomycin C-inactivated female D34 embryonic fibroblasts (‘BEF34’) served as feeders. Cells were seeded at 2.35×1^4^ cells/cm^2^ and cultured in Dulbecco's Modified Eagle Medium: Nutrient Mixture F-12 (DMEM/F12, Gibco) with Glutamax^TM^-I, 10% fetal calf serum (FCS, Invitrogen). This ‘somatic medium’ was supplemented with 2 mM valproic acid (VPA, Calbiochem). After seeding, cells were cultured for two days in somatic medium/VPA before transfection with 1 µg DNA per 2×10^5^ cells using lipofection (Lipofectamine^TM^ LTX/PLUS^TM^, Invitrogen) or nucleofection (programme A-24, Lonza, Germany). Two days post-transfection, lipofected cells were passaged onto 8-well glass chambers (BD, USA) or tissue culture dishes coated for 1 h at 2–4 µg/cm^2^ per tissue culture dish and 5–7 µg/cm^2^ per glass chamber with natural mouse laminin (Invitrogen). The next day, they were shifted into iPS medium (‘2i/LIF’), comprising of MEK1/2 inhibitor PD0325901 (0.4 µM, Stemgent, USA), GSK3B inhibitor CHIR99021 (3 µM, Stemgent), and human LIF (20 ng/ml, Genscript) in DMEM/F12 supplemented with N2 (Gibco) and mixed 1∶1 with Neurobasal medium (Gibco) supplemented with B27 (Gibco) and 1 mM L-glutamine (‘N2B27’) [14]. Nucleofected cells were seeded onto laminin-coated culture vessels in somatic medium/VPA and shifted into 2i/LIF N2B27 medium the following day. Culture medium was changed every 3–4 days.

### Colony tracking and size determination

Colonies were either passaged by mouth pipette-assisted dissociation into large fragments in accutase™ (Millipore, New Zealand) or by cutting with a splitting blade (ESE 020, Bioniche Animal Health, USA) mounted to a micromanipulator (MO-188, Nikon Narishige, Japan). Fragments were cultured in 2i/LIF and photographed at regular intervals. For size determination, brightfield images were imported into Image J (http://rsb.info.nih.gov/ij/) and areas measured using polygon selection.

### Cell proliferation assays

Real-time changes in cell number, viability, and morphology were quantified using an RTCA-SP xCELLigence™ system (Roche, New Zealand). For each treatment, cells were seeded in triplicate in 100 µl somatic medium at 2.35×10^4^ cells/cm^2^ onto laminin-coated 96-well E-Plates. In pilot experiments, the peak cell index (CI) for each cell line was determined. Each compound was diluted in pre-warmed medium and added at 1/4 to 1/3 of the peak CI, around 24 h after plating. CI readings were taken every 1 h. Curves were normalized on the respective CI values 30 min after compound addition when temperature of the fresh medium had equilibrated. Curve slope was determined during the interval between normalization time point and plateau phase (log phase), and normalized on the LIF-treated control.

DNA synthesis was assessed using a click-iT® EdU (5-ethynyl-2′-deoxyuridine) proliferation assay (Invitrogen). Cells were: fixed with 4% paraformaldehyde (PFA) every two days (‘pulse-fix’); washed out of EdU and cultured in medium supplemented with 10 µM thymidine quench (‘pulse-chase’); or kept in EdU (‘cumulative’). Cells stained without EdU labelling served as a negative control. In addition, we performed immunocytochemistry against Ki-67 and PCNA as described below.

### Detection of apoptosis

Apoptosis was examined with the click-iT® TUNEL Alexa Fluor® Imaging Assay (C10246; Invitrogen). Cells were fixed, permeabilized and stained according to the manufacturer's instructions. Cells stained without EdU-incorporation and DNase-treated cells provide negative and positive controls, respectively.

### DNA and RNA isolation

For genomic DNA isolation, cells or 50–100 mg of finely ground liquid nitrogen frozen tissue samples were lysed in 100 mM Tris pH 8, 200 mM NaCl, 5 mM EDTA, 0.1% SDS and 1 mg/ml proteinase K at 55°C. After 12–18 hours, samples were digested with RNAse A (10 µg/ml) at 37°C for 30 min, extracted twice with phenol/chloroform/isoamyl alcohol (25∶24∶1) and ethanol-precipitated. The air-dried pellet was resuspended in 50–100 µl H_2_O and used for PCR. For RNA isolation, cells or 50–100 mg of finely ground liquid nitrogen frozen tissue samples were lysed in TRIZOL® (Invitrogen)and cDNA synthesized as described [89]. Reverse transcriptase was omitted in one sample, each time a batch was processed for cDNA synthesis (‘-RT’).

### PCR and RT-PCR

A Mastercycler Gradient (Eppendorf, Germany) or Thermal cycler (Bio-Rad, New Zealand) was used for PCR amplification using primers shown in Table S1. Ectopic forward primers bind at different positions of vector-specific 5′-UTR, between pCMV and the first ATG of the insert (i.e. in pOSKM: pos. 618–711 for *OCT4*, pos. 2637–2738 for *SOX2*, pos. 4544–4631 for *KLF4* and pos. 6902–6995 for *c-MYC*). Ectopic reverse primers bind in their respective target gene. Endogenous primers span specifically between in the 5′-UTR (*SOX2*, *OCT4*) or 3′-UTR (*KLF4*, *c-MYC*) and the adjacent exon of their respective target gene. The PCR was performed using the following conditions: one cycle denaturation at 95°C for 5 minutes, followed by 35 cycles of 30 seconds at 95°C, 30 sec at 52–63°C (see Table S1 for primer-specific annealing temperatures), 30 seconds at 72°C; 7 min extension at 72°C and cooling to 4°C.

### Real-Time RT-PCR

A LightCycler® (Roche, New Zealand) was used for qPCR amplification and data analysis. All reactions were performed with the LightCycler® FastStart DNA MasterPLUS SYBR Green I Kit. Primers were designed using LightCycler® Probe Design 2.0 or NCBI/Primer-BLAST. The ready-to-use “Hot Start” LightCycler® reaction mix consisted of 0.4 ul of each primer (10 µM), 2.0 µl LightCycler® SYBR Green I master mix, 5.2 µl DEPC water, 1.0 µl DMSO if required and 1–2.0 µl cDNA template. The following four-segment program was used: 1) denaturation (10 min at 95 °C); 2) amplification and quantification (20 sec at 95 °C, 20 sec at 52–63 °C, followed by 20 sec at 72 °C with a single fluorescent measurement repeated 45 times); 3) melting curve (95 °C, then cooling to 65 °C for 20 sec, heating at 0.2 °C sec-1 to 95 °C while continuously measuring fluorescence); and 4) cooling to 4 °C. Product identity was confirmed by gel electrophoresis and melting curve analysis. For relative quantification, external standard curves were generated from serial 5-log dilutions for each gene in duplicate. One high efficiency curve (3.6≥ slope ≥3.1, R^2^>0.99) was saved for each target gene and imported for relative quantification as described [89].

### Alkaline phosphatase (AP) activity

Cells were washed with PBST (0.05% Tween® 20 in PBS), and fixed with 4% PFA for 2 min at room temperature. After PBST washing, cells were stained in 1 ml NTMT buffer (10 mM Tris pH 9.5, 100 mM NaCl, 50 mM MgCl_2_, 1% Tween® 20) with 3.38 µl/ml of 100 mg/ml NBT reagent (Roche) and 3.5 µl/ml of 50 mg/ml BCIP reagent (Roche) for 20 min in the dark. A commercial staining kit (Stemgent) was used occasionally.

### Immunoblotting

Western blot analyses were carried out using the following antibodies: phospho(Thr202/Tyr204)-p44/42 MEK1/2 (#9101) and p44/42 MEK1/2 (#9102, both Cell signalling). Whole cell lysates (40 µg per lane) were resolved on 4–12% Bis-Tris SDS-Page gradient NuPage gels, transferred to nitrocellulose membranes and blotted for phospho-MEK1/2 and pMEK1/2 (both 1∶1000). The secondary antibody, goat anti-rabbit IgG-HRP (Dako, P0448), was used at 1∶5000 and peroxidise activity was visualized with Western Lightning Plus-ECL kit (PerkinElmer, NEL105001EA).

### Immunocytochemistry

The following antigens were analyzed: SOX2 (AF2018, R&D Systems), OCT4 (sc-9081), SSEA-1 (sc-21702), SSEA-3 (sc-21703), and SSEA-4 (sc-21704, all Santa Cruz), Ki-67 (ab15580), PCNA (ab29), KLF4 (Ab72543), TRA-1-60 (Ab16288), and TRA-1-81 (Ab16289, all Abcam). Cells were fixed in 4% PFA for 15 min at 4°C, washed in PBS, quenched in 50 mM NH_4_Cl in PBS for 10 min, permeabilized in 0.1% (v/v) Triton X-100 in PBS for 10 min at room temperature and blocked in 5% donkey serum, 5% BSA in PBS for 30 min. Primary antibodies were incubated overnight at 4°C, washed in PBS and incubated with Alexa Fluor® 488 or 546 donkey anti-mouse, -rat, -rabbit or -goat secondary IgG antibodies (all Invitrogen) for 30 min at 38.5°C. All antibodies were diluted in blocking buffer. DNA was counterstained with 5 µg/ml Hoechst 33342 (Sigma). Preparations were washed in PBS and once in H_2_O before mounting (DAKO, Med-Bio Ltd., New Zealand). Negative controls were processed the same way, except that the primary antibodies were replaced with blocking buffer. Images were taken on an epifluorescence (Olympus BX50) or confocal microscope (Olympus FluoView FV1000).

### Telomerase activity

Telomerase activity was determined with the TRAPEZE® kit (Chemicon, USA). Heat-inactivated (85°C for 10 min) samples were used as internal negative controls. Reactions were separated on non-denaturing TBE-based 10% polyacrylamide (19∶1) gels, stained with SYBR Gold (Invitrogen) and visualized on a Gel Doc^TM^ 2000 documentation system (Bio-Rad).

### Karyotyping

Bovine iPSC-like colonies (D24 post-transfection) were cultured for 2 days in 2i/LIF with 10% serum replacer (Invitrogen), treated with 1.67 µM nocodazole (Sigma) overnight, trypsinized and centrifuged at 1000 rpm for 5 min. The pellet was resuspended in 0.56% KCl solution, incubated at 38°C for 15 min and fixed in −20°C methanol: acetic acid (3∶1) at 4°C for 30 min. Washing with fresh fixative was repeated twice before re-suspending the pellet in 500 µl of ice-cold fixative, spreading onto chilled microscope slides and staining with 10% KaryoMAX® Giemsa in Gurr buffer, pH 6.8 (BDH, New Zealand).

### Embryoid body (EB) formation

Undifferentiated iPSC-like colonies were picked by mouth pipette and cultured on bacterial-grade Petri dishes (Falcon, USA) for 5–21 days in N2B27. The medium was changed every 2 days.

### Teratoma formation

On D24 post-transfection, cells and colonies were harvested using a cell scraper, centrifuged and re-suspended in PBS +1% PVA (10–30k). Using a 23G needle, 100 µl of cell suspension (approximately 1–5×10^6^ cells per site) was injected intramuscularly into the quadriceps of adult immune deficient (SCID) male mice. After 5–7 weeks, tumors were graded [90], dissected, fixed overnight in Davidson's fixative, embedded in paraffin, sectioned, haematoxylin–eosin stained and analyzed by a pathologist service (Gribbles, New Zealand). Investigations complied with the New Zealand Animal Welfare Act 1999 and were approved by the Ruakura Animal Ethics Committee (AE Application 11849).

### Statistical Analysis

All values are presented as mean ± S.E.M, unless indicated otherwise. Statistical significance was accepted at P<0.05 and determined using the two-tailed t-test with equal variance (Fig. 1, 2, 4). Log ratios of the colony tracking data (Fig. 3) were analyzed using the residual maximum likelihood method in GenStat® (12^th^ Edition), with the treatments as fixed effects and individual colonies as random effects.

## Supporting Information

Figure S1 **Immunoblot analyses of steady-state levels of phospho(Thr202, Tyr204)-MEK1/2 and total MEK1/2 in BEF40 after 24h in N2B27 with solvent control (DMSO), PD at the indicated concentrations, 3 µM CHIR, 0.4 µM PD plus 3 µM CHIR (2i) or 2i/LIF.**
(TIF)

Figure S2 **Proliferation and apoptosis in bovine fibroblasts and iPSC-like cells (A) Non-transfected BEF40 cells were cultured in 2i/LIF or N2B27.** EdU+ nuclei were counted after EdU addition (solid blue horizontal lines) and fixation (solid blue vertical lines). BEF40 cells on D4 (B) and D18 (C) post-transfection with pKMOS were analyzed by immunofluorescence. DNA was counterstained with Hoechst 33342. White circles  =  positive PCNA/negative Ki-67; red circles  =  positive PCNA/Ki-67; arrowheads  =  negative PCNA/Ki-67. (D) Positive nuclei were counted at indicated time points. Open arrow indicates first emerging colonies. (E) Apoptotic nuclei were identified by Click-iT staining (arrows). DNA was counterstained with Hoechst 33342. Omission of EdU and DNase-treatment of cells provide negative and positive controls, respectively.
(TIF)

Figure S3 **iPSC-like cells express endogenous and ectopic iPS factors.** RT-PCR using primers specific for endogenous (A) and ectopic (B) *OCT4, SOX2, KLF4* and *c-MYC* mRNAs. cDNA was extracted from BEF40 one day post-transfection or from 10 pooled iPSC-like colonies 18–20 days post-transfection. cDNA from 50 bovine blastocysts/pool and pKMOS DNA provide controls. (C) PCR using plasmid-specific primers (*c-MYC, KLF4*). Genomic DNA was extracted from ∼150 colonies on D38 post-transfection. pKMOS and BEF DNA provide positive and negative controls; primers amplifying 18S DNA serve as loading controls.
(TIF)

Figure S4 **Molecular characterization of bovine iPSC-like colonies by confocal immunofluorescence.** DNA was counterstained with Hoechst 33342. Arrowheads indicate positive cells. Scale bar  = 50 µm.
(TIF)

Figure S5 **PCR analysis of genomic DNA and RT-PCR analysis of cDNA from bovine iPSC-like-derived teratomas.** Mouse ESC-derived teratomas provide a negative control. Species-specific primers amplify *ACTB* in bovine iPSC-derived (#1), but not mESC-derived teratomas, confirming bovine origin of tumour tissue. Primers amplifying both bovine and mouse 18S DNA and cDNA provide a loading control.
(TIF)

Table S1 Primers used for end-point and/or quantitative (q) real-time RT-PCR; * =  includes 4% or 10% DMSO for end-point or qPCR, respectively, ND  =  not determined.
(DOC)
