# Supplementary figures and images for: A Virus-Free Poly-Promoter Vector Induces Pluripotency in Quiescent Bovine Cells under Chemically Defined Conditions of Dual Kinase Inhibition
Source: PLoS One. 2011 Sep 2;6(9):e24501. doi: 10.1371/journal.pone.0024501 (PMC3166309; doi:10.1371/journal.pone.0024501)

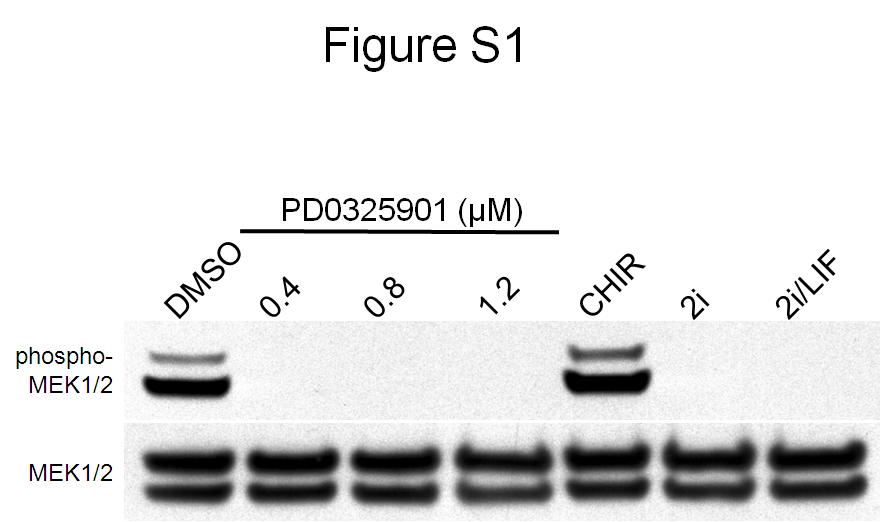

Supplement: Figure S1 — Immunoblot analyses of steady-state levels of phospho(Thr202, Tyr204)-MEK1/2 and total MEK1/2 in BEF40 after 24h in N2B27 with solvent control (DMSO), PD at the indicated concentrations, 3 µM CHIR, 0.4 µM PD plus 3 µM CHIR (2i) or 2i/LIF. (TIF) [file pone.0024501.s001.tif]

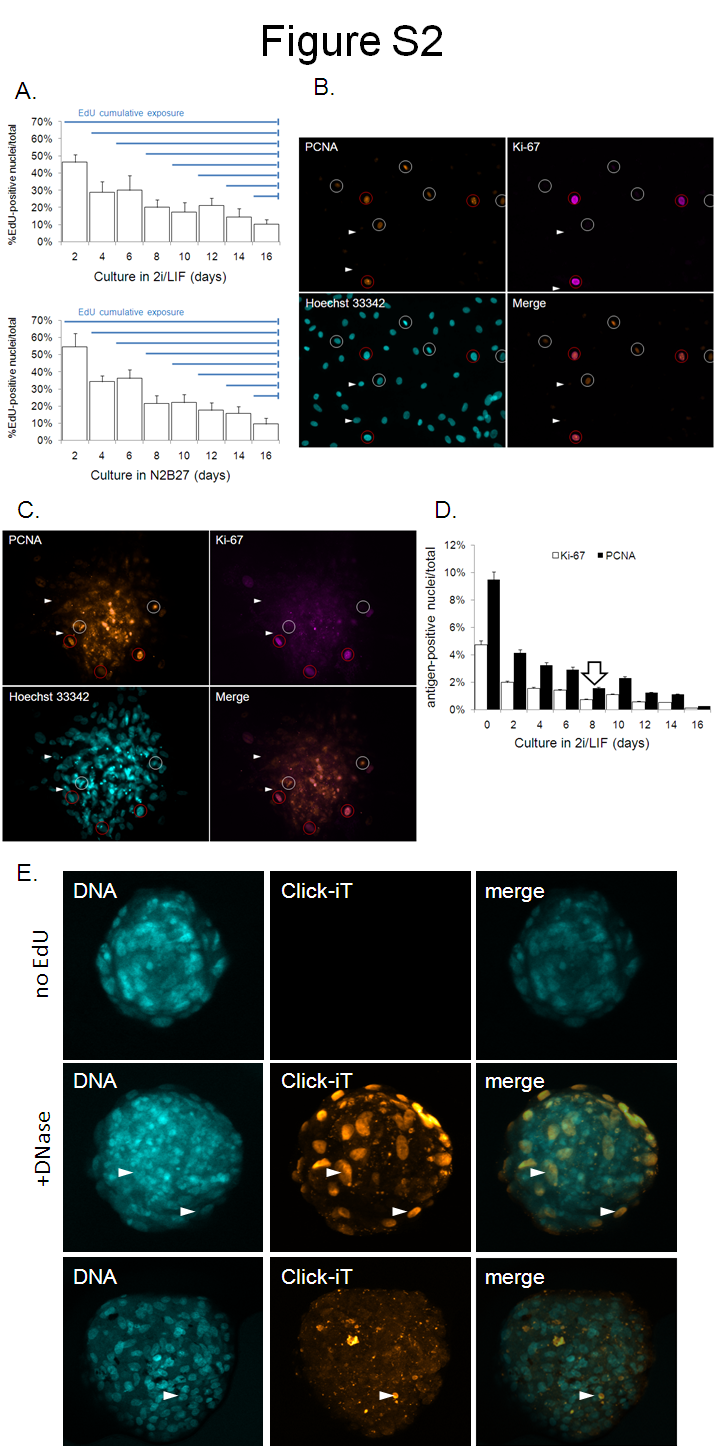

Supplement: Figure S2 — Proliferation and apoptosis in bovine fibroblasts and iPSC-like cells (A) Non-transfected BEF40 cells were cultured in 2i/LIF or N2B27. EdU+ nuclei were counted after EdU addition (solid blue horizontal lines) and fixation (solid blue vertical lines). BEF40 cells on D4 (B) and D18 (C) post-transfection with pKMOS were analyzed by immunofluorescence. DNA was counterstained with Hoechst 33342. White circles = positive PCNA/negative Ki-67; red circles = positive PCNA/Ki-67; arrowheads = negative PCNA/Ki-67. (D) Positive nuclei were counted at indicated time points. Open arrow indicates first emerging colonies. (E) Apoptotic nuclei were identified by Click-iT staining (arrows). DNA was counterstained with Hoechst 33342. Omission of EdU and DNase-treatment of cells provide negative and positive controls, respectively. (TIF) [file pone.0024501.s002.tif]

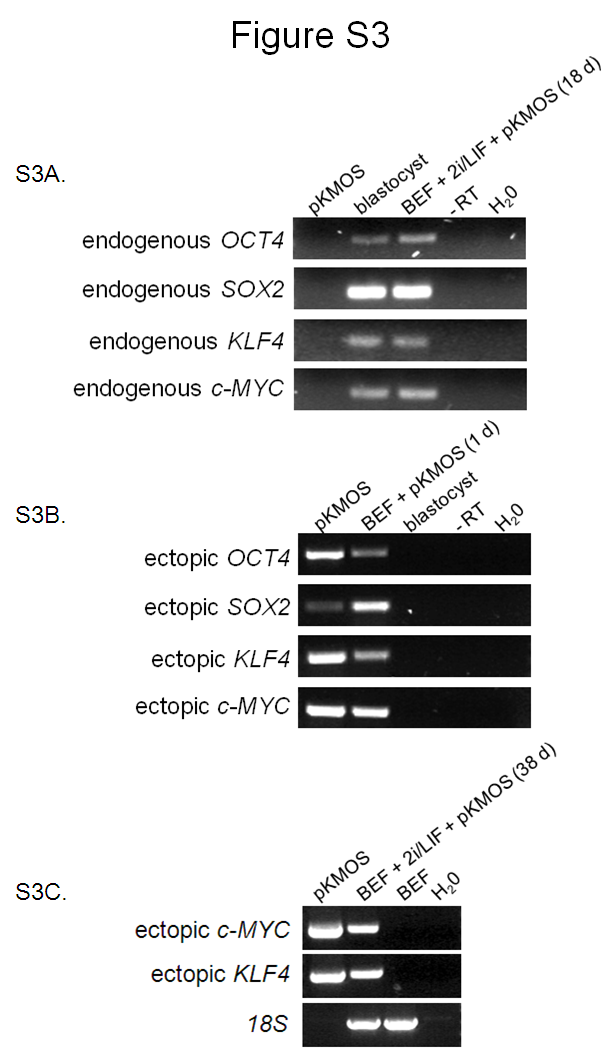

Supplement: Figure S3 — iPSC-like cells express endogenous and ectopic iPS factors. RT-PCR using primers specific for endogenous (A) and ectopic (B) OCT4, SOX2, KLF4 and c-MYC mRNAs. cDNA was extracted from BEF40 one day post-transfection or from 10 pooled iPSC-like colonies 18–20 days post-transfection. cDNA from 50 bovine blastocysts/pool and pKMOS DNA provide controls. (C) PCR using plasmid-specific primers (c-MYC, KLF4). Genomic DNA was extracted from ∼150 colonies on D38 post-transfection. pKMOS and BEF DNA provide positive and negative controls; primers amplifying 18S DNA serve as loading controls. (TIF) [file pone.0024501.s003.tif]

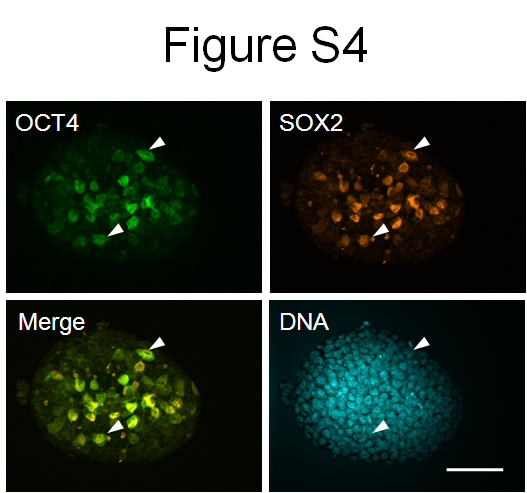

Supplement: Figure S4 — Molecular characterization of bovine iPSC-like colonies by confocal immunofluorescence. DNA was counterstained with Hoechst 33342. Arrowheads indicate positive cells. Scale bar = 50 µm. (TIF) [file pone.0024501.s004.tif]

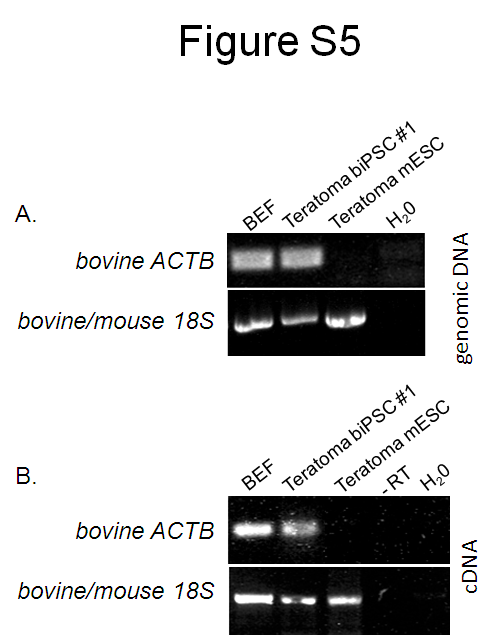

Supplement: Figure S5 — PCR analysis of genomic DNA and RT-PCR analysis of cDNA from bovine iPSC-like-derived teratomas. Mouse ESC-derived teratomas provide a negative control. Species-specific primers amplify ACTB in bovine iPSC-derived (#1), but not mESC-derived teratomas, confirming bovine origin of tumour tissue. Primers amplifying both bovine and mouse 18S DNA and cDNA provide a loading control. (TIF) [file pone.0024501.s005.tif]
