# Supplementary material for: A Virus-Free Poly-Promoter Vector Induces Pluripotency in Quiescent Bovine Cells under Chemically Defined Conditions of Dual Kinase Inhibition
Source: PLoS One. 2011 Sep 2;6(9):e24501. doi: 10.1371/journal.pone.0024501 (PMC3166309; doi:10.1371/journal.pone.0024501)
Supplement: Table S1 — Primers used for end-point and/or quantitative (q) real-time RT-PCR; * = includes 4% or 10% DMSO for end-point or qPCR, respectively, ND = not determined. (DOC) [file pone.0024501.s006.doc]

**Supporting Table S1**

| Gene |  | Sequence (5’- 3’) | RT-PCR annealing (°C) | qPCR melting peak (°C) | Amplicon size (bp) | Reference |
| --- | --- | --- | --- | --- | --- | --- |
| *OCT4** | F: | GGTTCTCTTTGGAAAGGTGTTC | 60 | 89 | 333 | this paper |
|  | R: | TGGCGACGGTTGCAAAACCA |  |  |  |  |
| *SOX2* | F: | CTATGACCAGCT CGCAGA | 58 | 86 | 152 | this paper |
|  | R: | GGAAGAAGAGGTAACCACG |  |  |  |  |
| *KLF4* | F: | TCCCACCGCTCCATTAC | 60 | 83 | 158 | this paper |
|  | R: | ATGAGAACTCTTCGTGTAGG |  |  |  |  |
| *c-MYC* | F: | GTGTCTACCCATCAGCACAATTA | 60 | 89 | 155 | this paper |
|  | R: | TTCTCCTCCGTGTCCGAA |  |  |  |  |
| ecto *OCT4** | F: | CATAGAAGACACCGGGACC | 58 | 90 | 500 | this paper |
|  | R: | GCTTGGCAAATTGTTCAAGG |  |  |  |  |
| ecto *SOX2** | F: | TAGTGAACCGTCAGATCGC | 58 | 90 | 268 | this paper |
|  | R: | GGGACCACACCATGAAGG |  |  |  |  |
| ecto *KLF4* | F: | GGGACCGATCCAGCCTC | 59 | 85 | 299 | this paper |
|  | R: | GATCATTGAACTCCTCCGTCT |  |  |  |  |
| ecto *c-MYC* | F: | CTGGAGACGCCATCCAC | 57 | 88 | 398 | this paper |
|  | R: | CTCCTAGTAGCTCGGTCAC |  |  |  |  |
| endo *SOX2* | F: | GTAGTTTGCTGCCTCTTTAAGAC | 60 | 90 | 153 | this paper |
|  | R: | CGCTTCCCTCCTCTTCTG |  |  |  |  |
| endo *KLF4* | F: | TCGGACCACCTCGCCTTA | 60 | 85 | 169 | this paper |
|  | R: | TTGTTGGGAACTTGACCATGAT |  |  |  |  |
| endo *c-MYC* | F: | AAGCTCAAGTCAGAAATAGACG | 60 | 89 | 157 | this paper |
|  | R: | TCCTCACTTTCCCTTAGTAACAAATG |  |  |  |  |
| endo *OCT4* | F: | GGCCAGAGGTCAAGGCTA | 60 | 83 | 218 | this paper |
|  | R: | ATCCAGGTCCGAGGATCA |  |  |  |  |
| *CDH1* | F: | GACACTGGAGGTATCAGCGCAC | 60 | ND | 194 | [1] |
|  | R: | TGATCTGGACCAGCGACTTAGG |  |  |  |  |
| *DPPA3** | F: | TGC AAGTTGCCACTCAACTC | 55 | 80 | 158 | this paper |
|  | R: | TCTTACCCCTCTCCGCCTAT |  |  |  |  |
| *NANOG* | F: | ATCCAGCTTGTCCCCAAAG | 56 | 87 | 438 | this paper |
|  | R: | ATTTCATTCGCTGGTTCTGG |  |  |  |  |
| *SALL4** | F: | CGGGTGCTCCAA TGAACTAT | 55 | 89 | 195 | this paper |
|  | R: | TGTCCTTCAAGATGA GCACG |  |  |  |  |
| *SOCS3* | F: | CCAGCCTGCGCCTCAAGACC | 60 | ND | 185 | this paper |
|  | R: | AAAGTGGCGCTGGTCCGAGC |  |  |  |  |
| *STAT3* | F: | GTGCATTGACAAAGACTCCG | 55 | ND | 200 | this paper |
|  | R: | AATCAGGGAGGCATCACAAT |  |  |  |  |
| *ZFP42* | F: | TGCCTGTCCTCACAACGGATGC | 60 | ND | 247 | this paper |
|  | R: | AGTGTGGGTGCGCACGTGTG |  |  |  |  |
| *FGF5** | F: | GCGACTTCCTCTTCTTCCC | 55 | ND | 151 | this paper |
|  | R: | GCAGATGGAAACCGATGC |  |  |  |  |
| *LEFTY2** | F: | CAGGGACTATGGAGCTCAGG | 55 | 92 | 268 | this paper |
|  | R: | CTGGATGCTGACAATCATGG |  |  |  |  |
| *T** | F: | CCAGTACCCCAGCCTGTGGTCC | 60 | 85 | 595 | this paper |
|  | R: | TGATGCCAGAGGCATCTCC |  |  |  |  |
| *MEF2C** | F: | AGCAAGAATACGATGCCATC | 60 | ND | 311/431 | [2] |
|  | R: | GAAGGGGTGGTGGTACGGTC |  |  |  |  |
| *GATA4** | F: | CTCGATATGTTTGATGACTTCT | 60 | ND | 346 | this paper |
|  | R: | CGTTTTCTGGTTTGAATCCC |  |  |  |  |
| *GFAP** | F: | CGGCACGAACGAGTCCCTGG | 60 | ND | 429 | this paper |
|  | R: | TGGGGCAGTGACCGCCAGAT |  |  |  |  |
| *TUBB3** | F: | CGGGGCCAAGTTCTGGGAGG | 60 | ND | 206 | this paper |
|  | R: | AGAGGTGCCCAAAGGCCCCA |  |  |  |  |
| *NES** | F: | GCAACTCCAGGCAGAGCGCA | 63 | ND | 836 | this paper |
|  | R: | TCAGCCCCCACAGTTGGCCT |  |  |  |  |
| *AFP** | F: | TGCAAGATGGCAGACCAGA | 56 | ND | 223 | this paper |
|  | R: | CCTGTGAGGCTATGACGGAA |  |  |  |  |
| *ACTB* | F: | GGCATCCTGACCCTCAAGTA | 52 | 86 | 100 | this paper |
|  | R: | CACACGGACCTCGTTGTAGA |  |  |  |  |
| *18 S rRNA* | F: | GACTCATTGGCCCTGTAATTGGAATGAGTC | 56 | 85 | 87 | [3] |
| (real-time PCR) | R: | GCTGCTGGCACCAGACTTG |  |  |  |  |
| *18 S rRNA* | F: | AACGTCTGCCCTATCAACT | 52 | ND | 699 | this paper |
| (end-point PCR) | R: | AACCTCCGACTTTGCTTCT |  |  |  |  |

**References**

1. Tesfaye D, Lonergan P, Hoelker M, Rings F, Nganvongpanit K, et al. (2007) Suppression of connexin 43 and E-cadherin transcripts in in vitro derived bovine embryos following culture in vitro or in vivo in the homologous bovine oviduct. Mol Reprod Dev 74: 978-988.

2. Nomura T, Ueyama T, Ashihara E, Tateishi K, Asada S, et al. (2008) Skeletal muscle-derived progenitors capable of differentiating into cardiomyocytes proliferate through myostatin-independent TGF-beta family signaling. Biochem Biophys Res Commun 365: 863-869.

3. Berg DK, Li C, Asher G, Wells DN, Oback B (2007) Red deer cloned from antler stem cells and their differentiated progeny. Biol Reprod 77: 384-394.
